# Properties and Microstructure Distribution of High-Performance Thermal Insulation Concrete

**DOI:** 10.3390/ma13092091

**Published:** 2020-05-01

**Authors:** Malek Mohammad, Eyad Masad, Thomas Seers, Sami G. Al-Ghamdi

**Affiliations:** 1 Division of Sustainable Development, College of Science and Engineering, Hamad Bin Khalifa University, Qatar Foundation, Doha 34110, Qatar; mmohammad@mail.hbku.edu.qa; 2 Mechanical Engineering Program, Texas A&M University at Qatar, Doha 23874, Qatar; eyad.masad@qatar.tamu.edu; 3 Department of Petroleum Engineering, Texas A&M University at Qatar, Doha 23874, Qatar; thomas.seers@qatar.tamu.edu

**Keywords:** lightweight concrete, high-performance concrete, thermal conductivity, expanded perlite, micro-computed tomography

## Abstract

The aim of this experimental study is to develop high strength and lightweight concrete mixture suitable for structural applications. This work investigates the effect of replacing normal aggregate either partially or totally with expanded perlite aggregate. This material allows for better thermal insulation properties, thus decreasing the energy usage within the life cycle of the concrete structure. Expanded perlite aggregate was used in concrete by 20%, 40%, 60%, 80%, and 100% in replacement of the natural aggregate. Material characterization tests of compressive strength, flexural strength, and thermal conductivity were carried out for six concrete mixtures. In addition, microstructure analysis was performed with the aid of a micro-computed tomography system to investigate the effects and relation of microstructure quantities on material properties. The proposed concrete mixture, which has 100% of expanded perlite aggregate, has a unit weight of 1703 kg/m^3^ and achieved reduction percentage of thermal conductivity around 62% (1.81 to 0.69 W·m^−1^·K^−1^) and a compressive strength of 42 MPa at 28 days; and thus is ideal for structural applications with enhanced properties.

## 1. Introduction and Background

Approximately one-third of total energy consumption is expended by the building sector, and this percentage is expected to reach 42.4% by 2030 [1,2,3]. In particular, the operational phase of buildings accounts for 73% of the building’s total energy and 64% of CO_2_ emissions [4]. It is widely accepted that energy production is the main source of greenhouse gasses emissions and is accordingly the main driver for climate change [5,6]. Previous research has shown that 60% of the energy consumed during the operational phase of buildings is used by heating, ventilation, and air conditioning (HVAC) systems [7]. Therefore, to decrease the overall negative environmental impact from the construction industry, it is imperative that attention be given to the materials used in building construction. In particular, the development of construction materials with better thermal insulation properties could greatly improve the energy efficiency of buildings [8].

It has been demonstrated that energy efficiency measures such as thermal insulation retrofitting are more cost-effective compared with the harvesting of renewable sources such as solar energy and wind energy [9,10]. Conventionally, thermal insulation of buildings was achieved mainly by incorporating insulation materials within the walls of buildings and increasing the thickness of building envelopes. However, this method increases the thickness of building walls, which is not desirable owing to space restrictions, expenses, transport volumes, architectural restrictions, and other limitations.

Because concrete is the most commonly used building material, researchers have explored methods for enhancing the properties of concrete materials [11,12,13]. Thermal conductivity, which is the ability of a material to transmit heat, is the property of main concern when evaluating concrete thermal properties. Concrete composed of commonly used aggregates has a thermal conductivity of 1.7–2.5 W·m^−1^·K^−1^, which is fairly high and thus exhibits poor insulation properties [14,15]. The thermal property of concrete is directly connected to its components and is thus based on aggregate type, aggregate proportion, moisture content, air voids, and any additional cementitious material. Consequently, significant effort has been expended to investigate the various aggregate types/proportions and their relationship with the concrete thermal properties [16,17].

In a previous study [18], the effects of varying the pumice aggregate-to-cement ratio within low-strength concrete were evaluated. The author reported that pumice concrete with a maximum ratio of 25:1 aggregate to cement is suitable for use as load-bearing blocks, although higher ratios are inadequate for industrial use in terms of strength and thermal conductivity. The effect of waste rubber incorporation in concrete was also analyzed in [19] and was found to decrease the thermal conductivity only slightly. Moreover, aerogel-incorporated mortar was evaluated for enhancing the thermal property of concrete [14]. It was found that a 50% volumetric incorporation of aerogel in a concrete mixture decreased the thermal conductivity only 0.55 W·m^−1^·K^−1^ but significantly decreased the compressive strength to 20 MPa. In an attempt to rectify such tradeoffs, significant research has been dedicated to investigating the use of perlite and expanded perlite (EP) material in concrete.

The popularity of perlite and EP is attributed mainly to its desired thermal conductivity properties and light weight. Perlite is a hydrated amorphous- volcanic silicate glass whose volume rapidly expands between 5 and 20 times of its original volume when heated about 900–1200 °C, forming EP [20]. The pore structure imbued upon EP during this expansion process provides EP with excellent thermal insulating properties and light weight when compared to common concrete aggregate lithologies (i.e., limestone, sandstone or granitic rocks), gaining the material great attention as a potential aggregate in concrete [21,22,23]. For example, the authors in [24] investigated the strength and thermal properties of concrete incorporated with EP filled with aerogel as a replacement for normal aggregate. In that study, graded and non-graded EP filled with aerogel were prepared separately at varying volumetric fractions. At 100% volume content of graded EP filled with aerogel (GEPA), the thermal conductivity of the concrete sample decreased to 0.098 W·m^−1^·K^−1^ although at the expense of a much lower compressive strength of 3.71 MPa. In addition, the effects of EP on the mechanical and thermal properties of lightweight concrete were tested in [25]. In that study, although the thermal conductivity decreased, the compressive strength was reduced substantially to 0.1 MPa with 100% replacement of aggregates with EP, and the unit weight of the concrete was decreased owing to the lighter weight of the EP. The authors in [26] evaluated the mechanical properties of self-compacted concrete with 20% of the cement replaced by fly-ash and the natural sand replaced by EP at various ratios (0–10% at 2.5% increments). The authors reported 26.2% decreased thermal conductivity with slightly 3.5% decreased compressive and 16.3% increase in flexural strength measurements. The authors of other research [27,28,29,30] applied comparable approaches, varying the relative proportion of EP aggregates in lieu of other aggregates and adjusting other cementitious proportions in the concrete mixture to achieve decreased thermal conductivity. Although the thermal properties achieved by such approaches is desirable in most of the works cited above, the decreased strength of the proposed mixtures rendered them unacceptable for structural use.

Although a significant amount of research has focused on the development of lightweight, thermally insulated concrete, the methods proposed in the literature have major limitations. First, most of the adjusted concrete mixtures have low mechanical strength, which renders them unacceptable for structural use. Second, the adjusted concrete mixtures with somewhat improved properties are suitable only for use in building envelopes such as blocks and plaster. The literature lacks the development of a concrete mixture that exhibits high mechanical properties and low thermal conductivity. Therefore, in the present study, we evaluate the influence of EP on the thermal and mechanical properties of high-performance concrete. The motivation behind this study is the development of a novel, lightweight concrete mixture that exhibits reduced thermal conductivity while retaining strong mechanical properties that enable its use in structural elements such as load bearing walls, columns, and roof slabs.

Motivated by the above, the work presented herein undertakes an experimental evaluation of an adjusted concrete mixture that incorporates various fraction of EP within the mix is conducted in this study. The reference concrete mixture is composed of ordinary Portland cement, microsilica, fly ash, river sand, and polypropylene microfibers. The replacement of sand with EP is applied in increments of 0%, 20%, 40%, 60%, 80%, and 100% of the volume of the sand. The resulting mixtures are tested to evaluate their mechanical properties such as compressive and flexural strengths, thermal properties such as thermal conductivity, and microstructural characteristics via X-ray computed tomography (CT).

## 2. Experimental Program and Methods

### 2.1. Materials and Mixture Design

For the experiments undertaken in this work, ordinary Portland cement, microsilica, fly ash, river sand with a maximum particle size of 2 mm, EP with a maximum particle size of 4 mm, and polypropylene microfibers with a length:diameter ratio of 12:0.18 mm were employed. The polypropylene microfibers were used to reduce the shrinkage and deformation in the plastic state. Polycarboxylic ether superplasticizer, provided by BASF, was used to maintain concrete workability with a low water/cement ratio (W/C).

**Figure 1:**
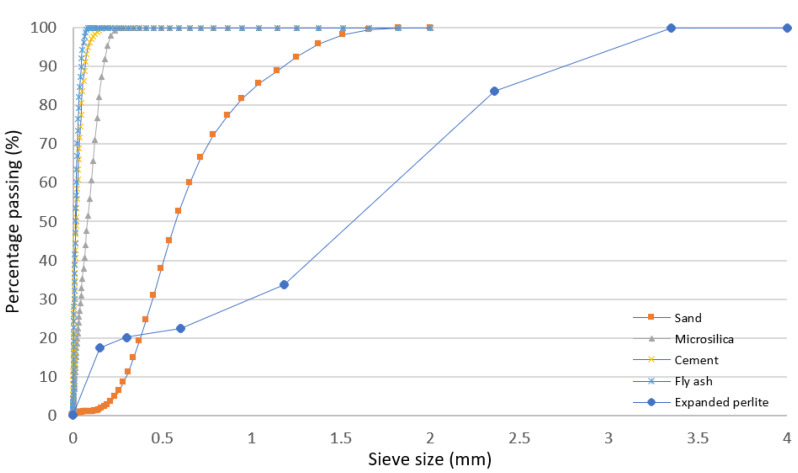
Grading curves of sand, microsilica, ordinary Portland cement, fly ash, and EP as a function of sieve aperture.

Particle size grading of the sand, cement, fly ash, and microsilica was conducted using a laser diffraction analyzer (LS 13 320, Beckman Coulter, Indianapolis, IN, USA). However, due to the large size of the EP aggregate (>2 mm), sieve analysis was implemented as per the ASTM C136/C136M-19 standard. Figure 1 illustrates the grading curves of all materials used in this work as a function of sieve aperture.

### 2.2. Experimental Procedures

#### Material Preparation

Six concrete mixtures were prepared with the same W/C ratio of 0.28 to test the effects of replacing normal aggregate with EP aggregate in increments of 0%, 20%, 40%, 60%, 80%, and 100% of the volume of the sand. The cementitious binder remained the same for all mixtures, containing 70%, 20%, and 10% of ordinary Portland cement, fly ash and microsilica, respectively. The manufacturer’s recommended dosages of superplasticizer and microfiber were used, at 1 L/kg of cementitious material and 1.2 kg/m^3^, respectively. Table 1 lists the materials used in all six mixtures and their respective proportions.

**Figure 2:**
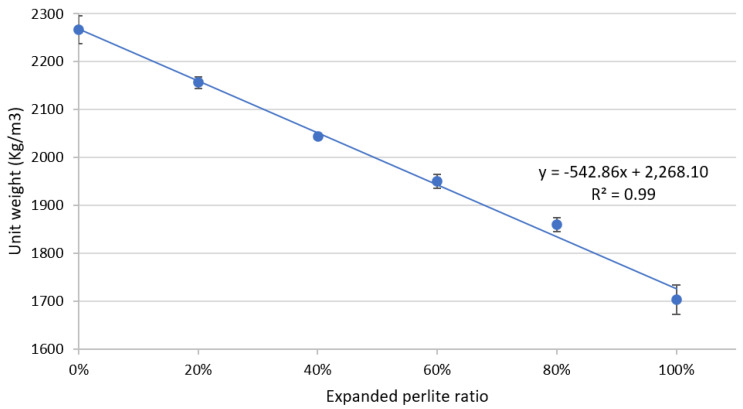
Unit weight (kg/m^3^) versus EP ratio.

Figure 2 illustrates the unit weight of each of the six mixtures as a function of the EP ratio contained in the mixture. The unit weight of the reference mixture was approximately 2270 kg/m^3^ and decreased approximately in a linear rate to 1700 kg/m^3^ for mixture 6 that has 100% EP.

### 2.3. Sampling

**Figure 3:**
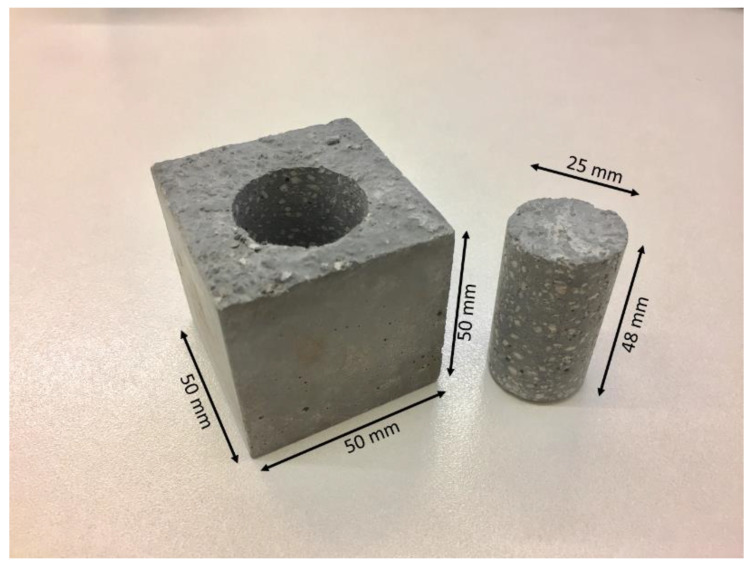
Illustration of sample preparation via coring for CT scan testing.

Fifteen samples were taken from each of the six mixtures for testing purposes. Twelve of these cubic samples, with dimensions of 50 × 50 × 50 mm, were used to test the compressive strength at different time intervals of 1, 7, 28, and 56 days. To evaluate the flexural strength, three beam specimens with dimensions of 40 × 40 × 160 mm were prepared and tested on day 28. Fragments that developed during the flexural strength testing were used for thermal conductivity analysis. One of the three remaining samples from the original 15 for mixtures 1, 4, and 6 was used for microstructural analysis using X-ray CT imaging. The samples were cored from the center of each 50 mm^3^ cube, resulting in a ~25.4 mm ø cylindrical sample with a length of ~50mm, as illustrated in Figure 3. CT imaging enabled enhanced analysis of the mixtures, and in particularly, for evaluating the internal structure of the material.

### 2.4. Testing

#### 2.4.1. Mechanical Properties Testing

The mechanical properties were tested according to ASTM standards. The compressive strength of three samples representing each mixture was tested on days 1, 7, 28, and 56 with a loading rate of 1200 N/s according to ASTM C109/C109M−16a. The arithmetic average was reported for each test. The flexural strength of each mixture was evaluated by testing three beam prisms with dimensions of 40 mm × 40 mm × 160 mm according to the ASTM C 348-18 standard on day 28.

#### 2.4.2. Thermal Conductivity Testing

**Figure 4:**
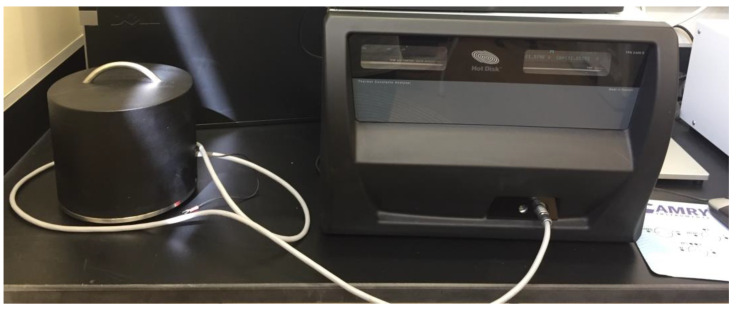
Hot Disk TPS 2500s thermal analyzer.

The thermal conductivity tests were performed on the flexural test specimens. The samples were heated for 48 h in an oven at a temperature of 60 °C and periodic weight measurements were taken to insure there was no change in the sample weight and thus has reached steady-state; indicated there was no more moisture remaining within the samples that may affect measurements [31,32]. The test was initiated by using a Hot Disk Thermal Analyzer (TPS 2500s) (Hot Disk, Göteborg, Sweden) with a disk-type Kapton sensor (5465) with a radius of 3.189 mm shown in Figure 4. This analysis technique used the transient plane source as per the ASTM D7984 [33] to measure the thermal conductivity, volumetric heat capacity, and thermal diffusivity. In particular, two samples from each mixture, each with a thickness of 40 mm, were used to cover both sides of the sensor. Heat was generated from an electric current passing through the sensor and was then transferred from the sensor to the samples at a rate depending on the thermal transport properties of that particular mixture [34]. The testing time was 5 min. The test was run twice for each mixture design; in each run, the analyzer was executed three times, thus yielding nine values for each design mixture.

#### 2.4.3. Microstructural Analysis

The microstructure of a material influences its physical, mechanical, and thermal properties [35,36,37]. In order to analyze the microstructural properties of the proposed concrete mixture, cross-sectional X-ray images were obtained from volume images of cylindrical samples cored from mixtures 1, 4, and 6, obtained using X-ray microcomputed tomographic (µCT) imaging. X-ray computed tomography is a non-destructive imaging technique, whereby volumetric (i.e., voxel) images are reconstructed from multiple axially acquired (2D) radiographic projections of the sample. Voxel images produced by X-ray computed tomographic reconstruction may be analyzed either directly as volumes or as axis aligned 2D images (orthoslices).

In this work, µCT images of concrete samples selected for microstructural investigations were captured using a Thermo-Fisher HeliScan (helical) (Thermo-Fisher Scientific, Waltham, MA, USA) micro computed tomographic scanner. The 25.4 mm diameter core samples were scanned using the maximum field of view of the system using space filling and large X-ray spot size modes, achieving a voxel resolution of 10.8 µm. The scanning procedure used an X-ray tube potential of 120 kV with a target current of 55 mA. A 0.1 mm steel shim filter was used on the X-ray tube diamond window to suppress beam hardening effects, with aluminum filters ranging between 4 mm (Mixture 6) and 6 mm (Mixture 1) thickness installed on the instrument’s flat panel detector. Samples were heated for 48 h in an oven at 60 °C to ensure that no moisture remained in matrix prior to scanning. Samples were then cooled to room temperature prior to scanning, to mitigate sample movement due to thermal contraction during image acquisition.

**Figure 5:**
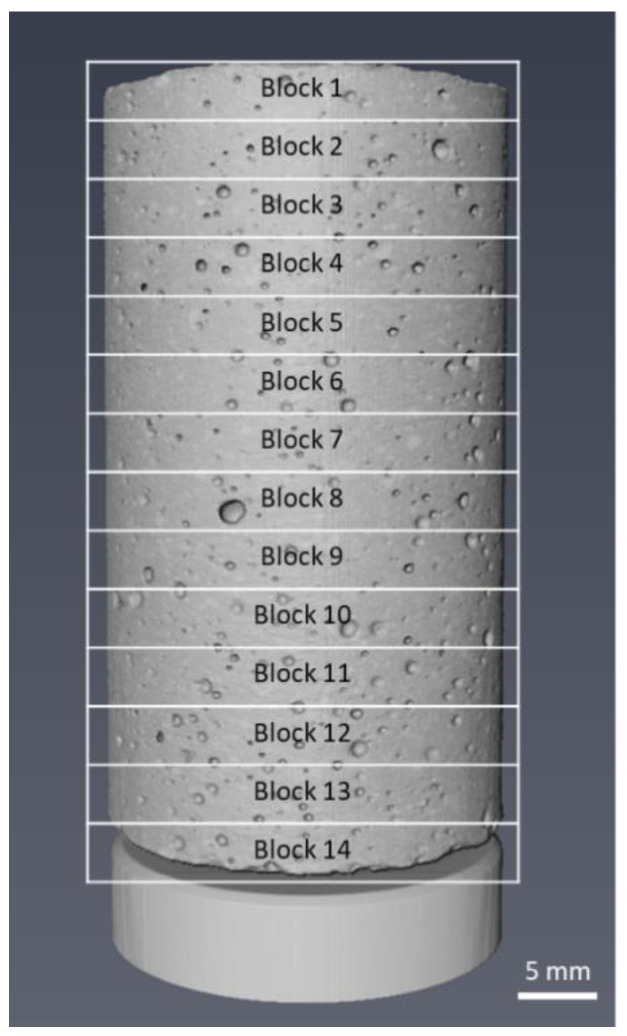
Reconstructed volume image mixture 1 concrete sample, captured using µCT imaging.

Microstructural analysis of the CT scans was performed using Thermo-Fisher Avizo Fire (Thermo-Fisher Scientific, Waltham, MA, USA) image analysis suite. In order to study the effect of EP addition in the concrete mixture upon concrete microstructure, the volume fractions of the cementitious material paste, air voids, and EP were measured. To reduce computational overhead, each concrete sample volume separated into 14 sub-volumes, each consisting of ~160 orthoslices (Figure 5).

The image analysis workflow was conducted separately on each block. Interactive thresholding was implemented to ensure clean segmentation between the paste material, the observable pore-space and EP content. Interactive thresholding is a gradient cut method, whereby the user visually determines grayscale cut off values to segment each material phase. After quality checking the results of the segmentation, quantitative image analysis was conducted to measure the amount of paste to air within each of the imaged concrete mixtures.

The imaged samples containing expanded perlite fragments offer considerable challenges in terms of macro-pore characterization using µCT imaging, with EP particulate having similar X-ray attenuation characteristics (and thus grayscale values) to air. In this work, blob detection, and sphericity analysis is used to segregate the two aforementioned material phases from µCT images. Sphericity is the degree to which an object conforms to a perfect sphere. Qualitative analyses of the control concrete mix (mixture 1) showed that the air voids exhibit highly were spherical geometries, with EP typically exhibiting a high degree of angularity and low sphericity, providing a practical criteria through which to segment the phases. To achieve this objective; label analysis was performed on the EP-air void segmented binary images to separate the blobs via their connected components. The sphericity of each disconnected blob was calculated as

(1) $$\Psi =\frac{\pi^{\frac{1}{3}}{\left(6V_{p}\right)}^{\frac{2}{3}}}{A_{p}}$$

where $V_{p}$ is the volume of the particle, $A_{p}$ is the surface area of the particle, and Ψ is the sphericity [38]. The volume and surface area, $V_{p}$ and $A_{p}$, were extracted from each image. Finally, the air voids were extracted from the labelled image using a threshold value of Ψ=0.78.

## 3. Results and Discussions

### 3.1. Compressive Strength

**Figure 6:**
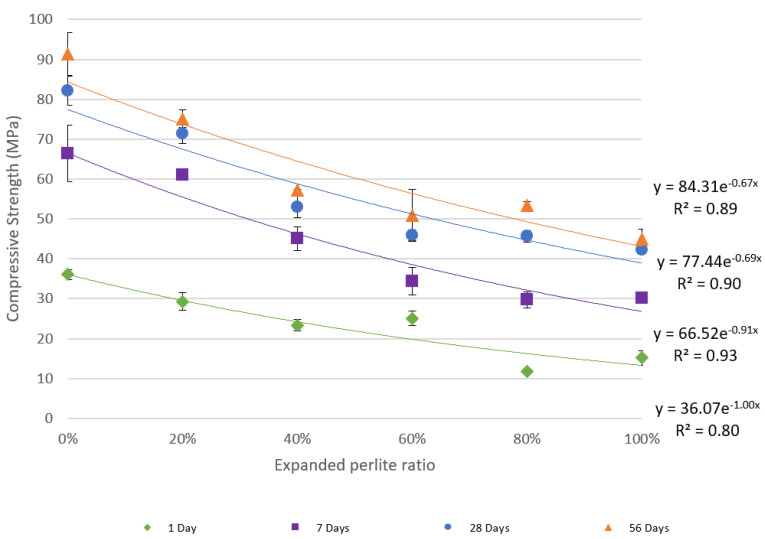
Effects of EP incorporation on the compressive strength of the cement.

The compressive strength testing results for each concrete mixture at days 1, 7, 28, and 56 are given in Figure 6. The results indicate a non-linear decrease in compressive strength as the amount of EP in the mix increases; an exponential function was found to fit the data well in terms of achieving the highest R^2^ values. For discussion purposes, the mixture with 0% EP is referred to as the reference mixture. The highest compressive strength, 91.4 MPa, was observed for the reference mixture on day 56. Two main observations were noted. First, as expected, the compressive strength of the concrete material decreased as the amount of EP present in the mixture increased. In particular, the largest decrease in compressive strength with respect to the reference mixture was noted at 20% and 40% EP. However, the decrease in compressive strength when the EP content increased from 60% to 80% and from 80% to 100% was not as significant when tested on days 28 and 56.

The decrease in compressive strength with increasing amounts of EP in the concrete mix can be explained by the low compressive strength of EP owing to its high porosity; therefore, as more amounts of sand are replaced by EP, the compressive strength of the mixture inherently decreases.

Despite this decrease in compressive strength, the resulting measurements are still acceptable and are comparable to the strengths of structural concrete mixtures. Figure 6 also shows relatively high coefficient of determination (R^2^) values, which indicate good exponential fitting to the data points. The error bars in the graph represent the standard deviation between the results for each reported average.

### 3.2. Flexural Strength

**Figure 7:**
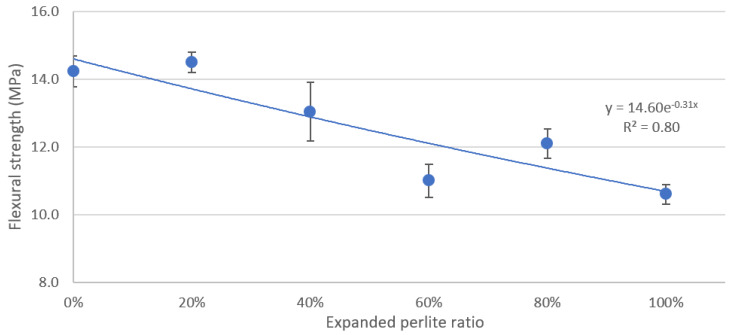
Flexural strength versus the percentage of EP in the mixture.

Figure 7 shows the flexural strength measurements for the six mixtures when tested on day 28. Similar to compressive strength data in Figure 6, an exponential function was used to fit the flexural strength data and it yielded an R^2^ of 0.80. The flexural strength of the concrete decreased from 14.2 MPa at 0% EP in the mixture to 10.6 MPa when 100% of the sand was replaced by EP.

### 3.3. Thermal Conductivity

**Figure 8:**
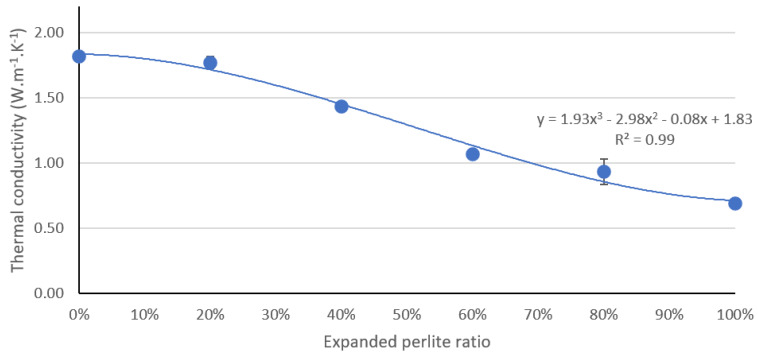
Effect of EP aggregate on the thermal conductivity of the concrete.

The thermal conductivity of a material is a measure of its ability to transfer heat. Typically, materials with higher levels of thermal conductivity (e.g., metals) are efficient for conducting heat, whereas the opposite is true for materials that exhibit lower levels of thermal conductivity (e.g., insulating materials such as Styrofoam). Porosity is a main factor that affects the thermal conductivity of concrete because the pores are filled with air, which has low thermal conductivity. The replacement of normal aggregate by EP increases the total porosity of the concrete and thus decreases the thermal conductivity and increases the thermal insulation of the concrete. This is illustrated in Figure 8, which outlines the tested thermal conductivity of all six mixtures. The gradual replacement of the aggregate by EP reduced the overall thermal conductivity from 1.9 W·m^−1^·K^−1^ at 0% EP to 0.69 W·m^−1^·K^−1^ at 100% incorporation of EP; thus, achieving a desirable 62% decrease in thermal conductivity.

### 3.4. X-ray µCT Analysis

**Figure 9:**
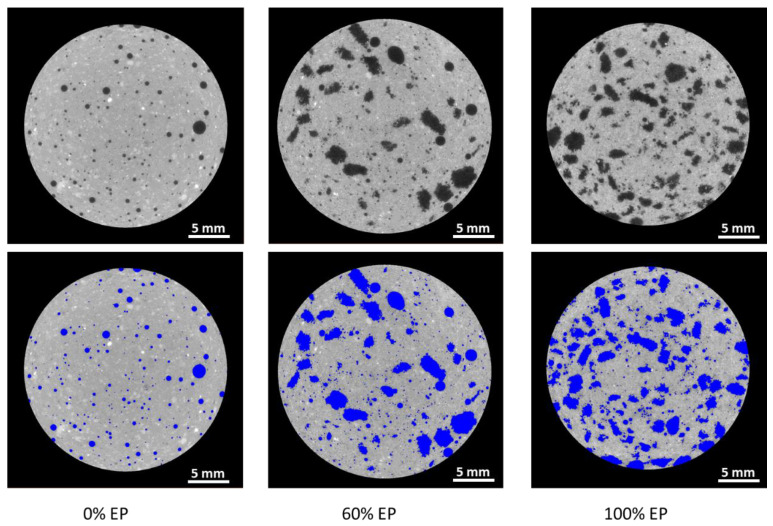
Cross-sectional CT image of block 7. (**Top**) Raw CT image scan. (**Bottom**) Thresholding applied to facilitate discrimination between paste particles and air voids/EP particles.

In this section, μCT analysis is performed for three of the mixtures, namely, mixtures 1, 4, and 6. Mixture 1 (0% incorporation of EP) was chosen as it is the baseline for comparing the other mixtures that incorporate EP. Mixtures 4 and 6 were chosen to demonstrate the properties of the concrete when 60% of the aggregate is replaced with EP (mixture 4) and when 100% of aggregate is replaced with EP (mixture 6). Interactive thresholding was applied to the images to segment the image into paste and air voids/EP particles. Figure 9 displays µCT scan xy orthoslices prior to (top images) and subsequent to interactive thresholding, with EP content within the sample set increasing from left to right (0%: left, 60%: center, and 100%: right). Note that lower grayscale values (darker areas) correspond to materials with relatively low X-ray attenuation (air/expanded perlite) and high grayscale values (grays to whites) represent more attenuating materials (i.e., concrete paste). As shown in Figure 9, at 0% EP content, air voids selected by the thresholding algorithm have a high degree of sphericity ($\overset{=}{µ}$ = 0.98/Table 2). It is clear from Figure 9 that in the case of samples incorporating expanded perlite, interactive thresholding cannot be used to isolate macro-pores within the concrete mixture, with the threshold value for both EP and air voids being broadly equivalent [39].

To segregate the air voids and EP in mixtures 4 and 6, with 60% and 100% EP incorporation, respectively, a geometric segmentation based upon sphericity was used. More specifically, image features with sphericity values equal to or less than 0.78 were labeled as EP, and image features with sphericity values over 0.78 were labelled as air voids. Table 2 shows the average sphericity, mean diameter, and volumetric fraction for mixtures 1, 4, and 6.

**Figure 10:**
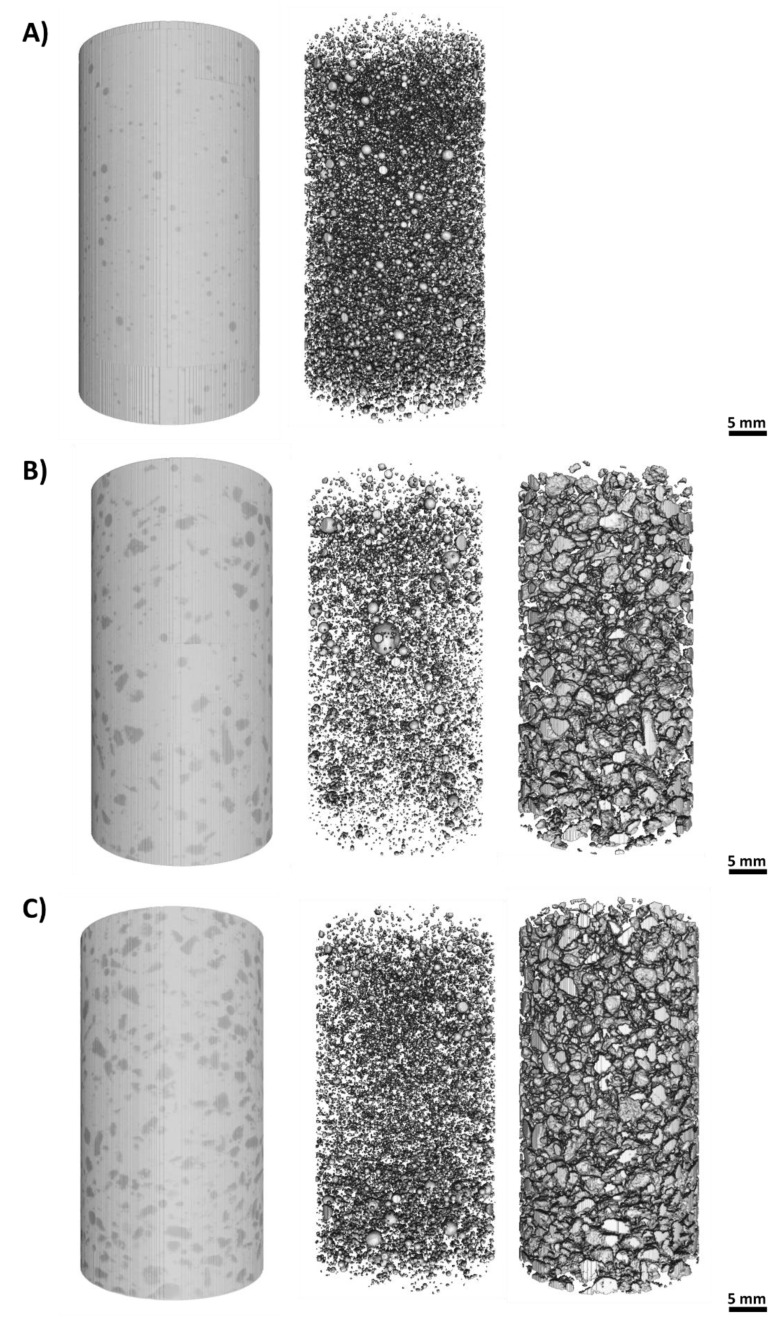
Illustration of binarization and segmentation process in (**A**) mixture 1 (0% EP), (**B**) mixture 4 (60% EP), and (**C**) mixture 6 (100% EP). The left-hand side of the panels show the volume rendering of the samples before binarization and segmentation; the middle images show the air voids; and the right-hand images show the EP aggregate.

Lightweight concrete may experience segregation between the aggregate and the paste material [17,40,41], thus resulting in non-homogenous concrete exhibiting undesirable qualities. 3D volume renderings of the samples for 0%, 60%, and 100% EP are shown in Figure 10. No material segregation between the EP aggregate and the paste material was evident within the samples, thus verifying the crucial homogeneity throughout the imaged volume.

**Figure 11:**
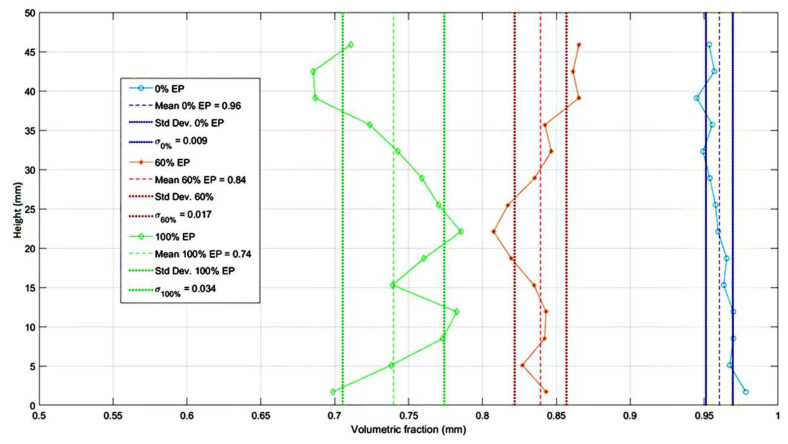
Volumetric fraction of paste throughout the sample’s height for mixtures 1, 4, and 6.

To corroborate results of the µCT image analysis presented herein, the data in Figure 11 represent the volumetric fraction of the paste throughout the sample depth. The coefficient of variation (CoV) which is defined in the following equation

(2) $$\mathrm{CoV}=\frac{\mathrm{standard}\;\mathrm{deviation}}{\mathrm{mean}}\times 100\%$$

which is a good parameter for comparing uniformity amongst the three studied material phases, was 0.94%, 2.0%, and 4.6% for mixtures 1, 4, and 6, respectively. Although the variation increased with an increase in the EP percentage, all values CoV relatively remained low, and the paste material was considered to be fairly uniform throughout the depth of the sample for all mixtures. Thus, the ratio of paste to the total sample volume per block was considered to be relatively uniform along the major axis of the core sample in each studied mixture. This suggests that in all cases, the distribution of the volumetric components of the concrete mixtures, and by extension the spatial distribution of their thermo-mechanical properties is relatively homogeneous.

### 3.5. Comparison of Proposed Concrete Mixture versus Published Results

**Figure 12:**
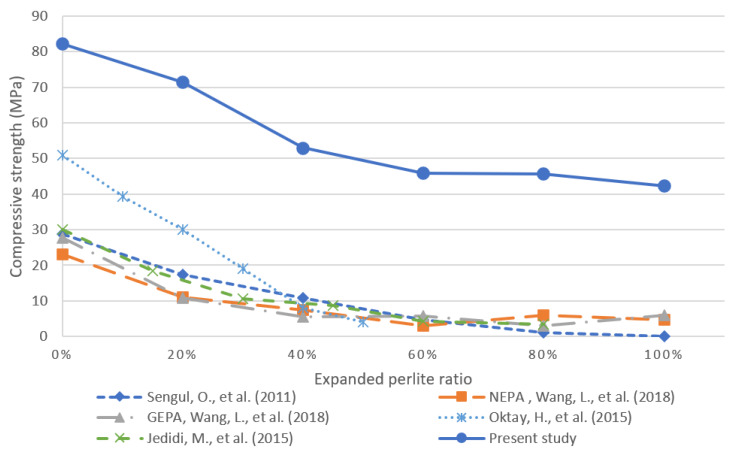
Comparison of the results of the present study and those reported in the literature for compressive strength as a function of the volumetric fraction of incorporated EP [24,25,29].

**Figure 13:**
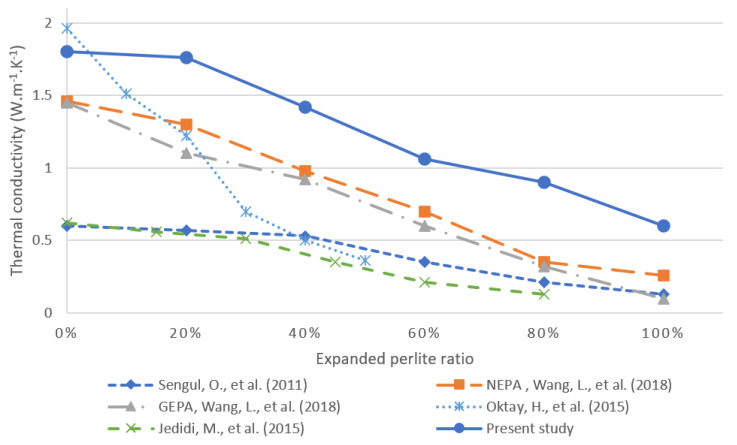
Comparison of the results of the present study and those in the literature regarding thermal conductivity as a function of the volumetric fraction of incorporated EP [24,25,29].

The replacement of normal aggregate (sand) by EP in concrete mixtures exhibited favorable properties, particularly in the significant decreases in thermal conductivity. Although the incorporation of EP did decrease the overall compressive and flexural strengths of the concrete, the decrease was not significant, and the resulting measurements were comparable to those reported in the literature and to measured values for standard industry-grade concrete. Therefore, the use of concrete containing EP is justifiable for structural elements. Figure 12 and Figure 13 compare the compressive strength and thermal conductivity, respectively, in several concrete mixtures reported in the literature against the proposed mixture in this work. The aim of these works is the reduction in thermal conductivity. The work in [25] investigates the effects of EP incorporation on the mechanical properties and thermal conductivity of lightweight concrete. Therein, the W/C ratio was kept constant at 0.55, and measurements were recorded for the gradual replacement of natural aggregate by EP.

The work in [24] investigated the use of EP filled with aerogel as a replacement for typical aggregate in concrete. In particular, the authors conducted an experimental study using graded EP filled with aerogel (GEPA) and non-graded perlite filled with aerogel (NEPA). The experiments were conducted with different volumetric replacement amounts of typical aggregate from 0% to 100% replacement in increments of 20%. The experimental study in [29] investigates a new cement-based material by incorporating various amounts of EP to replace the normal aggregate. Finally, the work in [30] evaluated the effect of EP on lightweight concrete at a W/C ratio of 0.7. Figure 12 shows that the proposed material outperformed all other studied materials in terms of compressive strength. In addition, the mixtures proposed in the present study have slightly higher thermal conductivities than those in the literature as shown in Figure 13, which can be attributed to differences in the unit weight and measurement method [42]. The proposed mixture obtained superior strength in comparison with previous studies due to introducing perlite to a reference/control mix that had compressive strength of 82.17 MPa at 28 days. This strength was achieved primarily by using different admixtures and a low w/c ratio. As a result, the high strength and low thermal conductivity makes this material appropriate for use in structural elements such as bearing walls, roof slabs and columns, while achieving improved insulation than conventional structural concretes. 

## 4. Conclusions

This paper introduces a lightweight but strong concrete mixture that uses EP to replace varying volumes of sand aggregate. The proposed mixture obtained superior strength in comparison with previous studies due to introducing perlite to a reference/control mix that had high compressive strength (approximately 80 MPa at 28 days). This strength was achieved primarily by using low W/C ratio and different admixtures especially micro silica.

The proposed mixtures showed high compressive and flexural strengths, making it suitable for use in appropriate structural elements with similar strength requirements. In addition, the low thermal conductivity of the proposed concrete makes it a promising material for use in buildings because the improved thermal insulation results in lower energy consumption for heating and cooling. Furthermore, the microstructural analysis via CT imaging revealed that the proposed lightweight concrete mixtures are relatively homogenous throughout the samples, which is critically important but uncommon in lightweight concrete.

Future research will focus on conducting life cycle assessment and life cycle costing to determine the sustainability benefits of using the EP mixtures developed in this study in various structural elements and buildings.

## Figures and Tables

**Table 1:** Proportions of materials used in the six mixtures.

Mixture Number	Mix 1	Mix 2	Mix 3	Mix 4	Mix 5	Mix 6
Expanded perlite percentage	0%	20%	40%	60%	80%	100%
River sand (kg/m^3^)	1241	992.8	744.6	496.4	248.2	0.00
Expanded perlite (kg/m^3^)	0	12.77	25.55	38.32	51.09	63.86
Cement (kg/m^3^)	579	579	579	579	579	579
Fly ash (kg/m^3^)	165	165	165	165	165	165
Microsilica (kg/m^3^)	83	83	83	83	83	83
Water (kg/m^3^)	232	232	232	232	232	232
Microfiber (kg/m^3^)	1.2	1.2	1.2	1.2	1.2	1.2
Superplasticizer (kg/m^3^)	8.27	8.27	8.27	8.27	8.27	8.27

**Table 2:** Size distribution and shape quantification of mixtures 1, 4, and 6.

Mixture Design	Mix 1: 0% EP	Mix 4: 60% EP		Mix 6: 100% EP	
Particle type	Air Voids	Air Voids	EP	Air Voids	EP
Mean sphericity ($\bar{\Psi })$	0.98	0.95	0.67	0.95	0.65
Mean equivalent diameter (mm)	0.39	0.23	0.73	0.24	0.76
Mean 3D volume (mm^3^)	0.07	0.03	0.56	0.02	0.57
Volume fraction %	1.45	1.15	7.78	1.07	9.99
